# The evolution of mental health outcomes across a combat deployment cycle: A longitudinal study of the Guam Army National Guard

**DOI:** 10.1371/journal.pone.0223855

**Published:** 2019-10-30

**Authors:** Dale W. Russell, Cristel Antonia Russell

**Affiliations:** 1 Consortium for Health and Military Performance, Department of Military and Emergency Medicine, Uniformed Services University of the Health Sciences, Bethesda, Maryland, United States of America; 2 Pepperdine University, Graziadio Business School, Malibu, California, United States of America; Stellenbosch University, SOUTH AFRICA

## Abstract

In the United States, National Guard soldiers have been called upon at unprecedented rates since 2001 to supplement active duty military forces. Frequent military deployments generate many occupational and environmental stressors for these citizen-soldiers, from serving in a dangerous zone to being away from family and home for long periods of time. Whereas there is a substantial amount of research focused on deployment-related health outcomes in relation to active duty (i.e., full-time) military populations, reserve forces are less understood. This study focuses on a United States Army National Guard combat unit deployed to Afghanistan. This prospective longitudinal study was conducted over the course an operational deployment cycle (i.e., before, during, and after) to document the evolution of salient mental health outcomes (i.e., post-traumatic stress, depression, general anxiety, and aggression). The findings show that both combat (e.g., killing others) and non-combat (e.g., boredom) stressors negatively affect mental health outcomes, and the severity of these outcomes increases over the course of a deployment cycle. Of special note, the study reveals key gender differences in the evolution of post-traumatic stress (PTS), depression, and anxiety across a deployment cycle: females report increased PTS, depression, and anxiety 6 months post-deployment, whereas the levels reported by males stabilize at their mid-deployment levels. The findings offer insights for medical providers and policymakers in developing more targeted health promotion campaigns and interventions, especially at the post-deployment phase.

## Introduction

### The evolution of mental health outcomes across a combat deployment cycle: A study of a Guam-based National Guard Unit

Despite the growth of research on military populations, the underlying factors and full implications of sustained contemporary military operations on the health and well-being of servicemembers are still not fully understood [1]. In relation to the United States (US) military, one shortcoming is that the extant literature has given more focus to the active component (AC; i.e., the full-time force) servicemembers, resulting in a knowledge gap concerning the heavily relied upon reserve component (RC; i.e., part-time force). In the US, the RC comprises the federally controlled Army Reserve (USAR) and the state controlled National Guard (NG).

The roots of the NG dates back to 1636, when citizen-soldiers formed militia units to defend their local communities [2]. The NG is a unique population within the Army as it is not only charged with providing the bulk of the RC’s combat forces (the USAR’s focus is on support forces) for military operations but it is also charged with supporting state government domestic missions, which range from responding to natural disasters, terrorist attacks, and supporting law enforcement during civil unrest [3–4]. Since the 9-11 terrorist attacks, the US military has been continually engaged in global operations which have placed an unprecedented level of pressure on the NG given numerous combat deployments, in addition to domestic missions [2].

As a result, the NG has been exposed to numerous unique stressors which have manifested into higher levels of mental health (MH) issues compared to their AC/RC peers [1, 5–8]. These outcomes are further frustrated by the fact that NG personnel often lack access to treatment options [9–10]. Despite such circumstances, the NG remains relatively understudied [11–12]; furthermore, little research has accounted for the unique cultural and socio-economic differences across the 54 states and territories, which could serve to inform health and well-being outcomes [13]. As such, this understudied population warrants research attention to identify risk and protective factors associated with its occupational exposures and enhance its health and well-being needs [11–12]. This research therefore seeks to improve our understanding of the risk/protective factors among NG SMs who are intermittently exposed to both military and civilian occupational exposures to help enhance their health and well-being [7, 14].

### Key health implications associated with military service

In the US, a large number of servicemembers and veterans continue to suffer MH and maladaptive behavioral issues despite the numerous programs that strive to mitigate the negative outcomes associated with military service [15–16]. Among the MH issues, Posttraumatic Stress Disorder (PTSD) remains a forefront concern and persists in military and veteran populations [17]. Military population PTSD rates are higher than those observed in the general population [18–20]. The core symptoms of PTSD include hyperarousal, avoidance, numbing, and re-experiencing of trauma [21–22]; its etiology is distinctive amongst other MH outcomes in that the defining traumatic event can usually be identified, thus allowing for a more precise cause-and-effect relationship [23–24]. Although PTSD is a key concern within military populations, it is often comorbid with other MH outcomes (e.g., depression and suicidal ideation) as well as problematic substance use [25–26]; for example, those with PTSD are seven times more likely to concurrently suffer from depression compared to those without PTSD [27–28].

Servicemembers face a variety of environmental stressors during a military deployment. One of the most documented stressors is combat exposure (e.g., fighting, killing/injuring others, threat to oneself, exposure to death/injury, and witnessing atrocities) known to trigger negative MH and maladaptive behavioral outcomes (e.g., substance abuse and misuse) [10, 29]. In addition to combat stressors, which can ebb and flow, servicemembers face a number of more constant deployment-related environmental stressors, which include boredom, tedious and monotonous workloads, poor food quality, a lack of personal time and space, inability to maintain hygiene, difficulty in maintaining social support relationships with family and friends back home, as well as interpersonal issues with their unit’s leadership [30–31, 8].

These issues are particularly salient for RC servicemembers who often struggle with the rapid transition between military and civilian life when both called up for and released from active duty [32–34]. For instance, reservists returning from a deployment often exhibit increased levels of maladaptive behaviors (e.g., aggressive behavior and substance use) and negative MH outcomes (e.g., depression and PTSD), whether or not they were exposed to combat [7, 35–37]. Despite the known outcomes associated with deployment-related environmental stressors, they have not garnered much research attention compared to direct combat stressors [38]. Yet, previous research shows that many non-combat deployment factors contribute to veterans’ ill health [39]. As such, a prospective longitudinal approach is needed to provide better insights concerning the relationship between how exposure military stressors, both combat and non-combat, evolve into MH and maladaptive behavioral issues over the course of a deployment cycle [40].

### Context and research question

The issues listed above are particularly salient in the context of this study, which was conducted with an infantry unit from the Guam Army National Guard. The US Territory of Guam is considered a strategic linchpin in regards to the US’s ability to project power in the Asia-Pacific region [41]. Guam is one of four inhabited Marianas Islands; the islands are administratively distinct in that Guam is classified as a US Territory while the other islands constitute a US Commonwealth. Guam was initially colonized by the Spanish but became a US possession after Spain’s defeat in the Spanish American War of 1898. Guam is noted as being more Americanized than the other Marianas Islands, likely due to close-nit political relationship with the US, which largely revolves around the island’s importance in hosting several large Navy and Air Force commands; indeed, Guam’s main economic stimulation comes from the US defense industry, followed by tourism [42]. The large presence of US military forces has not only impacted Guam’s economy stability but also its populace as a large number gravitate to military service [43]. Many from Guam join the military not only to as means for personal development (i.e., pay and benefits) but also to serve their country; additionally, following the brutal occupation of the island by Japan during World War II, serving in the US military has been a means by which to help protect the nationhood of Guam [44]. Culturally speaking, Chamorro men rely on military service to affirm their masculinity [44]. Indeed, the number of recruits joining the military was on the rise in Guam during the height of military operations in Iraq and Afghanistan, while the other states and territories were experiencing declines in recruiting [45].

Although Guam has had a militia dating back to 1771, while under Spanish rule, the current rendition of the NG stems from its establishment under US rule in 1917 [46]. The majority of this study’s participants were Chamorro (i.e., the indigenous people of the Mariana Islands). In part due to the large presence of the US military since World War II, there has been an inflow of non-natives to Guam; the population is now comprised of less than 50% Chamorro, with the current ethnic composition as follows: 37.3% Chamorro, 26.3% Filipino, 7.1% White, 7.3% Asian, 9% other Pacific Islander, 6% other Asian [42]. Although the Chamorro make up than half of Guam’s population, they comprise the majority of the Guam NG. While the focus on servicemembers from Guam may not generalize to the wider US or other military forces, this context represents a unique and understudied segment of the military in which to test the evolution of mental health issues over the course of a deployment and to assess how individual factors (e.g., gender and age) and experiences during deployment may affect these evolutions.

## Methods

This study utilizes a prospective longitudinal research design with data collected at three time points: pre-, mid- and post-deployment. Prospective longitudinal studies are suited to differentiate between short and long-term influences and serve to establish time-ordered associations between environmental exposures/stressors and health/behavioral outcomes compared to cross-sectional and retrospective studies [47]. This approach enhances the robustness of analyses by allowing an assessment of medical outcome trajectories. Unfortunately, this approach is seldom used in military studies due to logistical difficulties and resource limitations and, to our knowledge, it has not been undertaken before in such a manner with a NG cohort.

### Study approval

Study approval was obtained from the Institutional Review Boards (IRB) of the US Department of Defense’s (DoD) Uniformed Services University and the US Army Medical Research and Materiel Command. Additionally, as NG personnel fall under the control of the states and territories to which they are assigned, general study approval was also obtained from the National Guard Bureau and the unit’s Adjutant General (i.e., the overall commanding officer). For the deployment phase of the study, general study authorization was obtained from North Atlantic Treaty Organization and the US Central Command, which were responsible for servicemembers serving in Afghanistan.

### Study sample

The sample of reservists was drawn from an infantry brigade from the Guam Army National Guard that deployed to Afghanistan between April 2013 and January 2014. The brigade comprises 585 servicemembers, which represents ~48% of the territory’s entire force. Baseline pre-deployment survey data (N = 526; 89.9% of the unit’s total) were collected in April 2013 at the unit’s mobilization station several days before it deployed to Afghanistan. Data could not be collected from the entire unit prior to deployment as approximately 50 servicemembers had deployed ahead of the main unit to make preparations; however, these servicemembers were given an opportunity to partake in the remaining data collections. Mid-deployment survey data (N = 571; 97.6% of the unit’s total) were collected throughout Afghanistan in September and October 2013. Not all servicemembers were available due to mission requirements (e.g., on patrol); additionally, two servicemembers were killed in action prior to the mid-deployment data collection. Post-deployment survey data (N= 472; 80.7% of the unit’s total) were collected approximately six months post-deployment, when mental and behavioral health issues often develop [48–49]. Post-deployment data were collected at the unit’s primary home base in July and August 2014; however, some data were collected from servicemembers who were receiving medical care at Tripler Army Medical Center, Hawai’i in August 2014.

### Informed consent and data collection processes

For all data collections, servicemembers attended a recruitment briefing during routine duty hours that outlined the study’s purpose and their rights. During the briefing, servicemembers were encouraged to ask questions. Those wishing to partake in the study were required to complete an informed consent form at each time point; the consent forms were secured separately from the surveys to help maintain anonymity. Upon submitting their informed consent form, respondents were provided with a paper survey. Respondents were informed that they could skip any questions that made them uncomfortable and cease the study at any time. To help maximize the longitudinal study respondents’ confidentiality, surveys were de-identified using an anonymous self-generated sequential code (e.g., PPS1423) as to allow the linkage of individual respondent surveys across the three timepoints. To do so, as in previous military research, respondents listed the last character of their mother’s maiden name, day of their birth month, and year of their birth, the first digit of their birth month and the first letter of the city where they were born [50].

The surveys were matched with probabilistic record linkage based on the self-generated codes [51] in order to connect as many timepoints as possible. Given that this conservative matching process generated only 248 respondents matched at all three time points, the analyses adopted a multilevel modeling approach instead of the more constraining repeated measures approach.

### Survey measurements

Validated measures used in both previous military and civilian studies were administered at each of the three timepoints.

#### Mental and behavioral health measures

Post-traumatic stress: PTS symptomology was assessed using the 17-item PTSD Checklist (PCL-17; α= .98) [19, 52]. The PCL-17 lists all intrusion, avoidance, and arousal PTSD symptoms. Respondents rated each item on a 5-point scale (1 = *not at all* to 5 = *extremely)* and the sum of these responses provided an indicator of PTS symptom severity (17-item summation). Respondents with sum scores ≥ 50 were considered a positive screen for PTSD [19].

Depression: Depressive symptomology was assessed using the 9-item Patient Health Questionnaire subscale (PHQ-9) [53]. The sum of the 9 items provides an indicator of depression severity with probable major depression defined as endorsing five or more of the nine symptoms present “more than half the days” or “most days” in the past two weeks [53–54].

Generalized anxiety: Anxiety disorder symptomology was assessed with the 7-item Generalized Anxiety Disorder measure (GAD-7) [55]. The sum provides an indicator of severity and a score ≥ 10 was coded as a positive screen for anxiety.

Anger: Anger negatively impacts one’s ability to recover from traumatic experiences, especially following a military deployment [56–59]. Recent expressions of internally-focused anger were assessed using a measure adapted from the Interpersonal Conflict and the State/Trait Anger scales for use in military research [60]. Respondents indicated how many times in the past month (1 = *not at all* to 5 = *very often*) they had: boiled inside with anger, a hard time cooling down when angry, anger that got in the way of getting along with others, and anger that progresses instantly to aggression or rage (α = .93).

Aggression: Externally-focused aggressive behavior [61] was measured by having respondents indicate how many times in the past month (1 to 10+) they had: been angry at someone and yelled or shouted at them; been angry with someone and kicked or smashed something; slammed a door, punched a wall, etc.; threatened someone with physical violence; and gotten into a fight with someone and hit that person (α = .83).

General mental and physical health: Two single-item measures were used to assess respondents’ general mental and physical health. Respondents indicated the number of days over the past 30 days when they had poor mental or physical health.

#### Exposure measures

Deployment stressors: During the mid-deployment data collection, combat- and non-combat deployment stressors were assessed to capture for the degree to which each respondent experienced a number of deployment-related environmental stressors. Combat experiences place servicemembers at particular risk for developing a range of mental and behavioral health issues [10, 16, 19, 29] as well as physical ailments [20, 62]. Combat exposures were evaluated using a 31-item measure that has been used in multiple military studies (α = .78) [10, 19, 63]; respondents were asked to indicate how many times during their current deployment they had experienced each item (e.g., Being shot at; 0 = 0 to 6 = 5+). Non-combat deployment stressors were assessed with a 25-item measure created for the purposes of this study; respondents were asked to indicate how stressful (0 = *not at all* to 5 = *extremely*) each item had been on their current deployment (α = .95). This measure was developed specifically for this project to capture living conditions during deployment. It is similar in spirit to those used for veterans’ populations but focuses more squarely on current living and socio-emotional conditions. Scores ranged from 25 to 105 (Mean = 45.39, SD = 16.67) with a distribution skewed to the lower end of the scale (skewness = .89 and kurtosis = .26). It provided good internal consistency (α= .94). See S1 Appendix for a complete list of the items.

#### Additional variables

A number of factors known to impact both the onset and treatment of MH outcomes were also assessed in the post-deployment survey.

Organizational support: The degree to which respondents received positive psychological support from their unit overall was assessed with the shortened Perceived Organizational Support measure, which has been widely used in military research [64–65]; respondents indicated their level of agreement with the following items: 1) My unit strongly considers my goals and values; 2) My unit really cares about my well-being; 3) My unit cares about my opinion; and 4) My unit is willing to help me when I need a special favor (0 = *strongly disagree* to 5 = *strongly agree*; α = .95).

Unit cohesion: The degree to which respondents perceived their immediate unit as functioning cohesively was assessed by asking respondents to indicate their level of agreement with the following statements: 1) The members of my unit are cooperative with each other; 2) The members of my unit know that they can depend on each other; and 3) The members of my unit stand up for each other (0 = *strongly disagree* to 5 = *strongly agree*; α = .96) [66–67].

Facets of perceived leadership: Respondents’ perceptions of their immediate leadership in their unit (e.g., those directly in charge of them) were assessed by respondents indicating how often leaders did the following: 1) Tell servicemembers when they have done a good job; 2) Embarrass servicemembers in front of other servicemembers; 3) Try to look good to higher-ups by assigning extra missions or details to servicemembers; 4) Exhibit clear thinking and reasonable action under stress. Each of these items was measured from 0 = *never* to 5 = *always*) [68–70].

Reintegration: General post-deployment reintegration was assessed with a shortened 11-item version of the Military to Civilian Questionnaire [71]; respondents indicated the level of difficulty they had with each item (e.g., Finding meaning or purpose in life) since returning from the deployment (0 = *no difficulty* to 5 = *extreme difficulty*; α= .95). Additionally, post-deployment family reintegration was assessed with a shortened 10-item Army Post-Deployment Reintegration Scale [72]; respondents indicated the extent to which each item (e.g., I feel closer to my family) held true for them since returning from deployment (0 = *not true at all* to 5 = *completely true*; α= .78). See supplemental information for a complete list of the items.

Demographics: Consistent with other military studies, the key variables included: gender, military rank, age, ethnicity, education level, marital or significant other status, and years of military service. As this population is comprised of reserve personnel, more civilian-centric questions were also posed, including: civilian employment status (full-time, part-time, unemployed, retired), employment type (self-employed, government employee, or private sector employee), socioeconomic status (annual household income and debt level), and college/university student status (full or part-time). No personally identifiable information was collected (e.g., birthdate).

## Results

### Sample demographics

The demographics of the sample at mid-deployment are reported in Table 1. The majority of the 571 soldiers in the brigade were male (90.7%), Pacific Islander (84.6%), and Chamorro (i.e., native people of Guam; 80.80%). Ages ranged between 19 and 57 years at mid- deployment, with a mean age of 29.50. The majority (81.3%) had not deployed before so this was their first deployment.

**Table 1:** Demographic characteristics of the sample.

	N (%)
**Demographics** (as reported mid-deployment)	
N	571
Gender N males (% of total reported)	521 (91.7%)
Ethnicity N Pacific Islander (% of total reported)	450 (82.0%)
Age Mean (SD)	29.18 (8.52)
**Rank**	
E1-E3	37 (4.7%)
E4	298 (37.5%)
E5-E6	154 (19.4%)
E7-E9	34 (6.0%)
Officer / Warrant Officer	46 (5.8%)
**Education** (as reported pre deployment)	
< 12^th^ grade	10 (1.9%)
High School diploma / GED	267 (51.9%)
Some college / technical school	190 (37%)
Bachelor’s degree	37 (7.2%)
Graduate degree	10 (1.9%)
**Marital Status** (as reported pre deployment)	
Single never married	195 (37.4%)
Married	294 (56.3%)
Divorced or separated	33 (6.3%)
**Deployment History** (as reported pre deployment)	316
0 previous deployment	257 (81.3%)
1 previous deployment	32 (10.1%)
2 + previous deployments	27 (8.6%)

### Mental health outcomes

Table 2 provides the scores for each MH outcome of interest at the three timepoints and, where applicable, the total number and percentage of servicemembers who screened positive at each timepoint. There is a clear increase of positive screens for PTSD, depression and anxiety between pre- and mid-deployment and between mid- and post-deployment.

**Table 2:** Mental health outcomes across time points.

	Range of scores/scale	Pre-deployment	Mid-deployment	Post-deployment
Sample Size		N = 524	N = 559	N = 463
**Mental Health Outcomes**				
**Post-Traumatic Stress Disorder**				
PCL-17 Sum Score (SD)	17-84	23.89	28.24	28.97
PTSD Positive screen N (%)		28 (5.3%)	65 (11.4%)	66 (14.3%)
**Depression**				
PHQ-9 sum score (SD)	0-27	1.60	3.15	3.54
Depression Positive screen		8 (1.5%)	19 (3.3%)	35 (7.6%)
**General Anxiety Disorder**				
GAD-7 Sum Score (SD)	0-14	2.11	3.13	3.16
Anxiety positive screen N (%)		11 (2.1%)	29 (5.1%)	41 (9.1%)
**Internal Anger**	1-5	1.42	1.66	1.76
**Aggression**	0-10	.56	.65	1.39
**Bad Mental Health days (# in last 30 days)**	0-30	.37	1.06	3.70
**Bad Physical Health days (# in last 30 days)**	0-30	.85	1.78	2.14

Multilevel modeling was used to assess the evolution of the MH outcomes over the three timepoints and test the role of combat and deployment-related stress factors, as well as gender and age. This longitudinal mixed-effect model specification includes a time variable that represents each data collection point (baseline= 0) to account for changes in the outcome variables [73]. Linear mixed effects models were estimated using the nlme package [74] in the open source statistical programming language R [75]. Each of the continuous MH outcomes was analyzed in two steps. In the first model, only the main effects were included: time, gender, age, combat experiences and deployment stressors. In the second model, all the interactions with time were added. The analysis provides an assessment of how MH evolves across time (main effect of time) as well as whether these evolutions differ significantly as a function of the other variables (interaction between time and the other variables in the model).

As reported in Table 3, the main effects model shows that time was significant for all outcomes, reflecting a worsening of PCL-17, PHQ-9, and GAD-7 over the course of the deployment cycle but improvements in internal anger and aggression over time. Table 3 also reports the Akaike Information Criterions (AIC) and Deviance Information Criterions (DIC), which, along with R^2^ reflect model fit. These deviance indicators show that, for each outcome, the addition of the two-way interaction terms with time improves model fit. When comparing the main effects model to the full model, it is noteworthy that only the deployment-related stressors, and not combat-related stressors, remain significant predictors of all the MH outcomes. The full model, which includes the interactions, reveals that several predictor variables interact significantly with time, as discussed below.

**Table 3:** Estimates for the multilevel models predicting each mental health outcome.

	Mental Health Outcome				
	PCL-17	PHQ-9	GAD-7	Internal Anger	Aggression
**Main effects only model**					
Intercept	-0.44	-6.14**	-3.92**	0.97**	0.32
Time	2.54**	0.89**	0.54**	-0.16**	-0.37**
Gender	2.61 *	0.89*	0.65*	0.01^a^	0.10
Age	0.14 **	0.04**	0.02*	0.0008	0.002
Combat Experiences	0.15 **	0.03**	0.03**	0.006*	0.01**
Deployment Stressors	0.31 **	0.10**	0.09**	0.02**	0.02**
**Marginal R**^**2**^	0.26	0.22	0.26	0.18	0.12
**AIC**	9735.4	7125	6337.6	2993.3	4381.4
**DIC**	9693	7056	6262.9	2887.8	4288
**Deviance**	9706.2	7082.5	6292.2	2932.5	4326.7
**Full model (including interactions)**					
Intercept	13.31**	-0.52	-0.37	0.23	-0.90
Time	-4.79*	-2.10**	-1.36*	0.18	0.21
Gender	-0.86	-0.31	-0.67	0.40*	0.55
Age	0.02	-0.06 ^a^	-0.02	0.005	0.01
Combat Experiences	0.11 ^a^	0.001	0.02	0.004	-0.003
Deployment Stressors	0.18**	0.08**	0.07**	0.02**	0.03**
Time X Gender	1.84 ^a^	0.64	0.71*	0-.18*	-0.21
Time X Age	0.06 ^a^	0.05**	0.02 ^a^	-0.002	-0.005
Time X Combat Experiences	0.02	0.01	0.006	0.001	0.007^a^
Time X Deployment Stressors	0.07**	0.01	0.01^a^	-0.002	-0.005*
**Marginal R**^**2**^ **(DELTA)**	0.27 (.01)	0.24 (.02)	0.27 (.01)	0.19 (.01)	0.13 (.01)
**AIC (DELTA)**	9735.8 (.4)	7132.4 (7.4)	6356 (18.4)	3028 (34.7)	4410.3 (28.9)
**DIC (DELTA)**	9657.9 (-35.1)	7012.6 (-43.4)	6225.7 (-37.2)	2845.1 (-42.7)	4248.6 (-39.4)
**Deviance (DELTA)**	9684.8 (-21.4)	7060.5 (-22)	6278.9 (-13.3)	2924.6 (-7.9)	4317.4 (-9.3)
**Deviance Chi-Square (main effects vs full model)**	p= 0.0003	p= 0.0002	p= 0.01	p= 0.09	p= 0.05

Note: *p =< .05;

**p =< .01;

^a^ p =< .10

**Fig 1:**
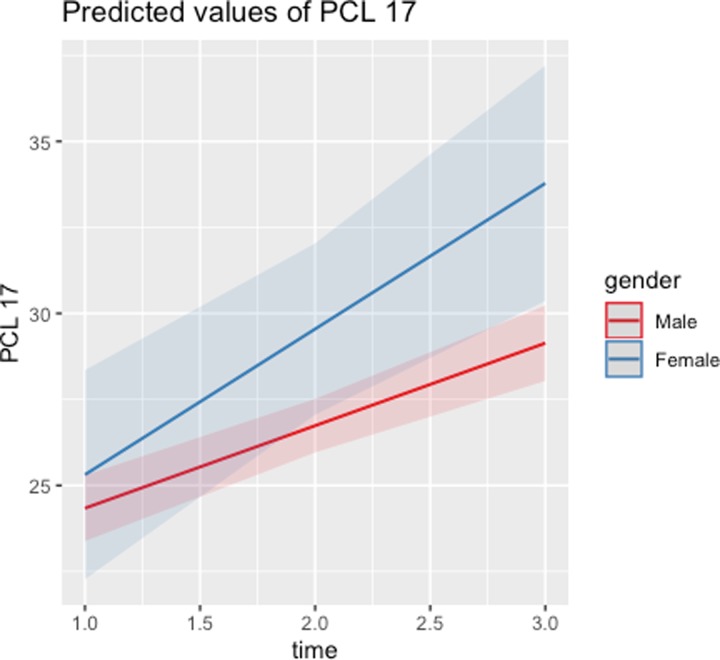
Predicted values of PCL-17 across time: Males vs. females.

**Fig 2:**
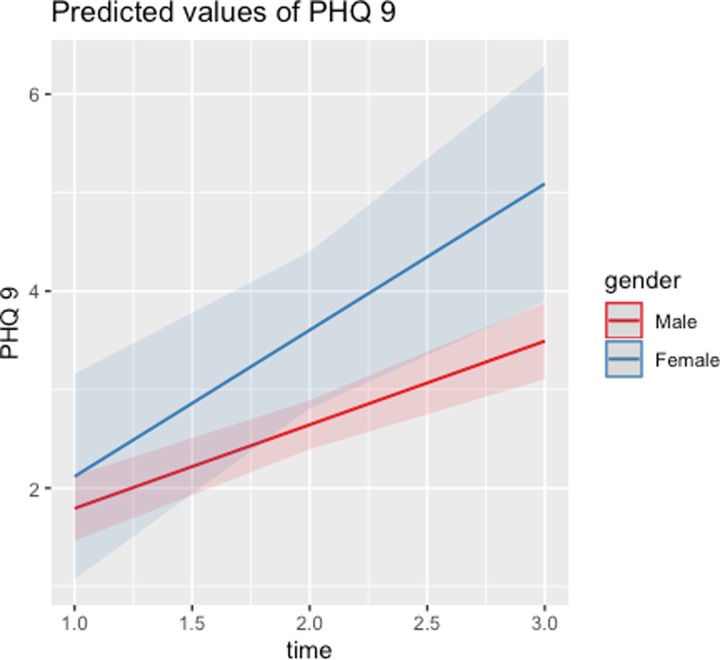
Predicted values of PHQ-9 across time: Males vs. females.

**Fig 3:**
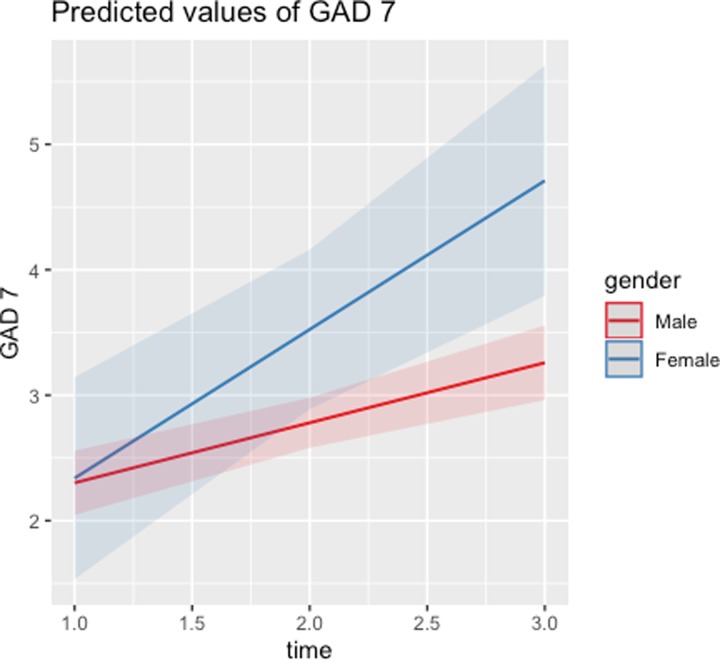
Predicted values of GAD-7 across time: Males vs. females.

Although there are no main effects in relation to gender, the interaction between time and gender is significant for GAD-7 and internal anger, and marginally significant for PCL-17. These interactions signal that the temporal evolution of these MH issues differs by gender. As illustrated in Figs 1–3, scores on the PCL-17, GAD-7, and PHQ-9 assessments stabilize around mid-deployment levels for males, but they are all significantly higher for females than for males six months post-deployment. Table 4 shows that female respondents report worse post-deployment MH issues, even though there were no such gender differences before or during deployment. Given that these analyses control for combat and non-combat deployment stressors, the gender differences identified are independent of those factors.

**Table 4:** Gender differences in mental health outcomes at each time points.

	Pre-deployment	Mid-deployment	Post-deployment
**Post-Traumatic Stress Disorder**			
PCL-17 Sum Score (SD) - Males	23.89 (10.61)	28.16 (12.71)	28.39 (15.60)
PCL-17 Sum Score (SD) - Females	24.00 (10.65)	29.27 (14.39)	35.44 (18.61)**
**Depression**			
PHQ-9 Sum Score (SD) - Males	1.57(3.18)	3.13 (4.50)	3.31 (5.46)
PHQ-9 Sum Score (SD) - Females	1.91 (3.68)	3.42 (4.56)	6.00 (6.94)**
**General Anxiety Disorder**			
GAD-7 Sum Score (SD) - Males	2.11 (2.79)	3.13 (3.43)	2.99 (3.76)
GAD-7 Sum Score (SD) - Females	2.02 (2.88)	3.29 (4.25)	5.02 (4.71)**

Note: significant difference between genders *p < .05;

**p < .01

**Fig 4:**
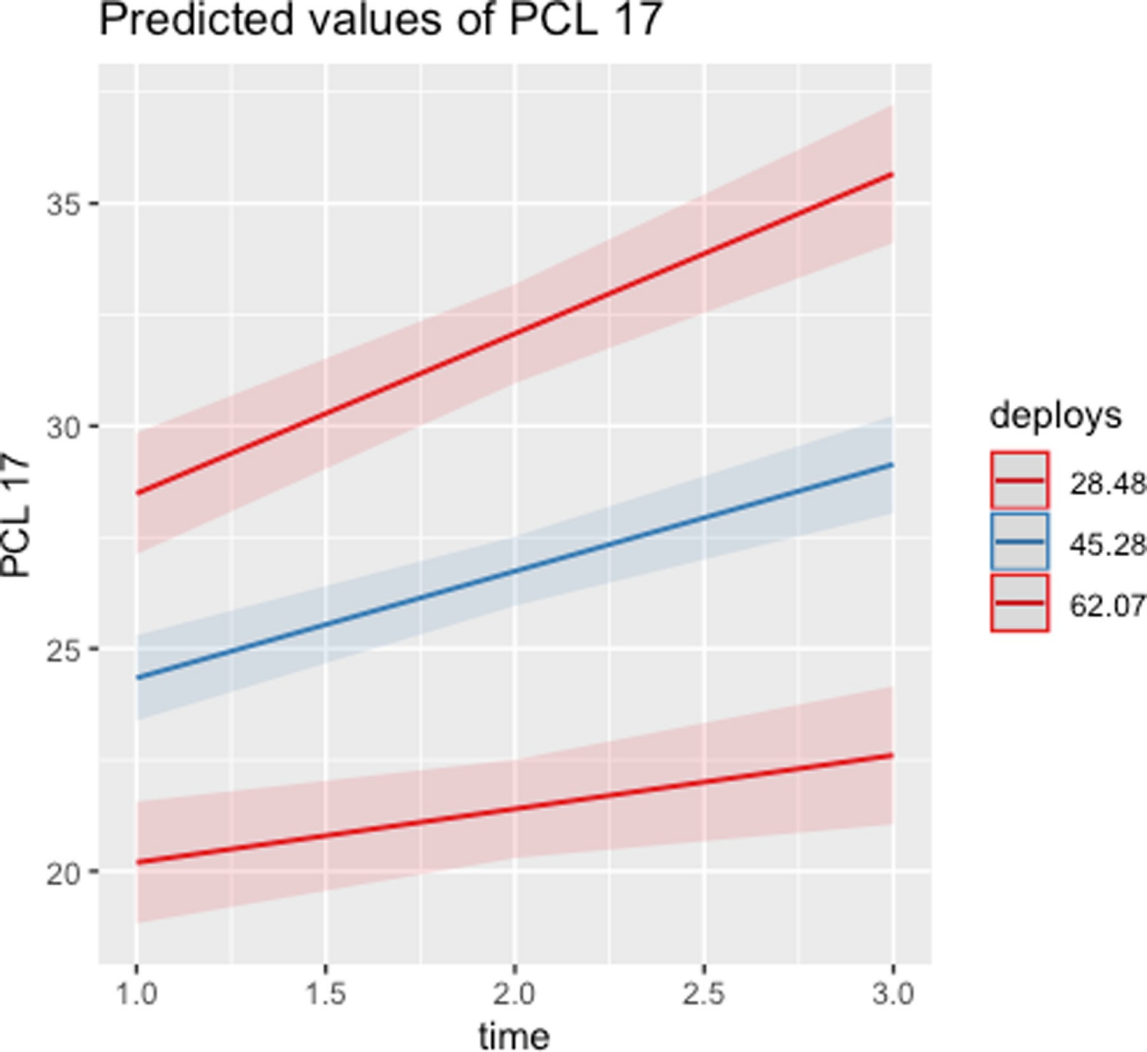
Predicted values of PCL-17 across time as a function of deployment stressors.

The multilevel modeling results further indicate that deployment stressors, not combat exposures, are significantly and positively associated with MH outcomes. The significant time X deployment stressors interaction also reveals that PCL-17 worsens at a greater rate over time for those with higher levels of deployment stressors, as illustrated in Fig 4.

### Exploratory post analysis

Given the significant gender differences in how MH symptoms evolve across the combat deployment cycle, an additional exploratory analysis was conducted to better understand the nature of the situation females face post-deployment. Focusing on the post-deployment data and comparing gender in an independent sample t-test between the two groups, females were found not to differ from their male counterparts in terms of family reintegration. Males and females did differ in their post-deployment assessment of the organization support received from the unit, with females reporting significantly lower rates of organization support (3.34 for females vs. 3.84 for males; *t* (461) = 3.47, *p* < .01), unit cohesion (3.55 for females vs. 4.00 for males; *t* (461) = 3.27, *p* < .01), and perceived unit leadership (2.95 for females vs. 3.24 for males; *t* (454) = 3.19, *p* < .01) even though there were no such gender differences at mid-deployment. These patterns are in the opposite direction of that observed in overall perceptions of general post-deployment reintegration where females reported higher levels than males (2.12 for females vs. 1.68 for males; *t* (461) = -2.50, *p* < .01). Hence, the worsening of female MH post-deployment appears to be connected to the perceived lack of support from their military unit, and not from factors related to their civilian lives.

## Discussion

This study reveals that the evolution of MH issues varies as a function of the type of stressor (i.e., both deployment and combat related) experienced across a deployment cycle as well as one’s individual characteristics. Unlike the bulk of prior research, the evidence in this study points to deployment stressors, and not combat-related stressors, as the major driver of the onset and continuation of MH issues. Although most of the extant literature has focused on combat-related stressors, this study’s findings suggest that more attention should be placed on everyday deployment-related stressors, which emerged as significant drivers of MH issues. Compared to combat-related stressors, non-combat stressors span a large spectrum and range from less serious factors, such as boredom, to more serious factors, such as sexual harassment [38, 76]. As to better account for the multitude of environmental stressors that servicemembers face while deployed, future research should more accurately identify, operationalize, and account for non-combat deployment-related stressors as to assess the degree to which they negatively impact health and well-being outcomes across a deployment cycle for active duty and reserve servicemembers alike.

Most notably, the findings highlight salient gender differences in how PTSD, depression and anxiety manifest post-deployment. Whereas females reported lower rates of MH issues before and during their deployment, their MH outcomes post-deployment are more severe than their male counterparts’. This finding adds to a growing body of research concerning gender-associated health outcome differences in military populations and highlights the need for additional gender-focused research [77]. To that end, an interesting avenue for future research might lie in more deeply documenting the factors, such as social support from fellow unit members, that may be lacking in the post-deployment environment for female servicemembers. Building on prior research that points to interpersonal stressors as particularly salient for females during deployment [78], this research signals that such stressors may also accentuate in the post-deployment period and female reservists’ transition back to their civilian lives. Considering that the females in this study reported higher rates of PTSD, depression and anxiety, as well as perceptions of receiving less unit and leadership support post-deployment, it is important that future research delineate what factors might underlie such perceptions and how to best palliate these cultural and organizational issues. Such findings are even more concerning when coupled with the fact that females are more likely to experience non-combat related interpersonal stressors (e.g., harassment and sexual assault) [79] which in turn leads to worse MH outcomes and negative career implications [80–82]. Additionally, female servicemembers are often faced with such interpersonal stressors while in the midst of a combat deployment, which can compound the negative outcomes [83]. These realities warrant additional research to better understand and help mitigate such outcomes, especially as the US military transitions to have female servicemembers serve in combat positions traditionally reserved for males [84–87].

Within such a line of research, a broader scope of inquiry is required to capture everyday realities post-deployment. It is well-documented that females face hardships and MH issues when returning to work following maternity leave. Research in this realm has shown the importance of accounting for socioeconomic status, childcare responsibilities, and household obligations [88]; and there have been calls for military researchers to better assess specific demographic, social, and environmental exposure factors [89]. Future research should increase focus to such factors when studying military reserve populations and assess the parallels between females returning from military deployments and returning to employment from parental leave. More specifically, future research should seek to understand and develop female-centric post-deployment reintegration and health promotion programs in an effort to mitigate negative MH outcomes.

This study helps narrow the gap in Guam-based academic research in general and in the military in particular. The focus on a Guam NG unit sheds light on an understudied subpopulation of the US military. In particular, this study provides insights on an isolated island nation that possesses strong and unique cultural identities and social bonds. This context highlights many of sociocultural identity facets that may affect one’s sense of meaning, purpose and belonging (e.g., being Chamorro, a member National Guard, male, American). Extending beyond this study, it would be useful to unearth aspects of the sociocultural environment that comprise risk factors, such as race-related stigma [90], as well as those that may afford protective factors, such as cultural belongingness Future research should seek to assess the interplay of these identity frames on the evolution of mental health and to guide the development of culturally relevant prevention and intervention efforts in the area of mental health [91].

## Supporting information

S1 File Deidentified minimal dataset used in the MLM analyses. (XLSX)

S1 Appendix Non-Combat deployment stressors. (DOC)
