# Supplementary material for: The evolution of mental health outcomes across a combat deployment cycle: A longitudinal study of the Guam Army National Guard
Source: PLoS One. 2019 Oct 30;14(10):e0223855. doi: 10.1371/journal.pone.0223855 (PMC6821079; doi:10.1371/journal.pone.0223855)
Supplement: S1 Appendix — (DOC) [file pone.0223855.s002.doc]

**S1 Appendix**

**Non-Combat Deployment Stressors**

Thinking about your current deployment, how stressful have the following experiences been?

(0= *not at all A little*, 1= *a little bit*, 2= *moderately*, 3= *quite a bit*, 4= *extremely*)

1. Being separated from family
2. Family problems back home
3. Difficulties communicating back home (e.g., by phone, email, or mail)
4. Boring and repetitive work
5. Uncertain redeployment date
6. Lack of privacy or personal space
7. Financial concerns
8. Not having the right equipment or repair parts
9. Not getting enough sleep
10. Continuous operations (i.e., operational tempo)
11. Long deployment length
12. Quality of the food
13. Not being able to stay clean
14. Lack of entertainment
15. Quality of the gym facilities
16. Lack of time between missions (i.e., reset time)
17. Quality of sleeping quarters
18. Interruptions while trying to sleep (e.g., noise levels)
19. Parenting concerns back home
20. Lack of personal time
21. End of a romantic relationship (e.g., divorce or break-up with significant other)
22. Enough time to sleep between missions /duty
23. Infidelity (i.e., cheating) of spouse / significant other
24. Trusting Allies (i.e., partner military personnel or local police)
25. Conflict (e.g., arguments) with spouse /significant other

**Post-Deployment Family Reintegration Scale [1]**

Rated from 1- not at all true to 5- completely true

- I feel closer to my family
- Putting the events of the tour behind me has been tough
- There has been tension in my family relationships
- I feel the tour has had a negative impact on my personal life
- I have become more involved in my family relationships
- I feel my family has had difficulty understanding me
- It has been hard to get used to being home again
- Getting back into sync with my family life has been hard
- Being back home has been a bit of a culture shock
- I more fully appreciate the time I spend with my family

**General post-deployment reintegration [2]**

Rated from 1- no difficulty to 5- extreme difficulty

- Feeling like you belong in 'civilian' society
- Making new friends
- Getting along with relatives
- Doing what you need to do for work or school
- Taking care of your chores at home
- Taking care of your health
- Enjoying or making good use of free time
- Taking part in community events or celebrations
- Dealing with people you do not know well
- Confiding or sharing personal thoughts and feelings
- Finding meaning or purpose in life

1. Blais AR, Thompson MM, McCreary DR. The development and validation of the Army post-deployment reintegration scale. Mil Psychol. 2009 Jun 30;21(3): 365-86.

2. Sayer NA, Frazier P, Orazem RJ, Murdoch M, Gravely A, Carlson KF, et al. Military to civilian questionnaire: A measure of postdeployment community reintegration difficulty among veterans using Department of Veterans Affairs medical care. J Trauma Stress. 2011 Dec;24(6): 660-70.
