# Supplementary material for: Correction: The evolution of mental health outcomes across a combat deployment cycle: A longitudinal study of a Guam-based National Guard unit
Source: PLoS One. 2020 Mar 4;15(3):e0230140. doi: 10.1371/journal.pone.0230140 (PMC7055881; doi:10.1371/journal.pone.0230140)
Supplement: S1 File — (PDF) [file pone.0230140.s001.pdf]

PLOS ONE

RESEARCH ARTICLE

The evolution of mental health outcomes across a combat deployment cycle: A longitudinal study of a Guam-based National Guard unit

Dale W. Russell[1]*, **Cristel Antonia Russell**[2]

1 Consortium for Health and Military Performance, Department of Military and Emergency Medicine, Uniformed Services University of the Health Sciences, Bethesda, Maryland, United States of America, 2 Department of Marketing, Pepperdine University, Graziadio Business School, Malibu, California, United States of America

* dale.russell@usuhs.mil

Check for updates

OPEN ACCESS

Citation: Russell DW, Russell CA (2019) The evolution of mental health outcomes across a combat deployment cycle: A longitudinal study of a Guam-based National Guard unit. PLoS ONE 14 (10): e0223855. https://doi.org/10.1371/journal.pone.0223855

Editor: Soraya Seedat, Stellenbosch University, SOUTH AFRICA

Received: April 12, 2019

Accepted: September 30, 2019

Published: October 30, 2019

Copyright: This is an open access article, free of all copyright, and may be freely reproduced, distributed, transmitted, modified, built upon, or otherwise used by anyone for any lawful purpose. The work is made available under the Creative Commons CC0 public domain dedication.

Data Availability Statement: All relevant data are within the paper and its Supporting Information files.

Funding: The second author acknowledges research support provided by the European Union's Marie- Sklodowska-Curie Actions. The funders had no role in study design, data collection and analysis, decision to publish, or preparation of the manuscript.

Competing interests: The authors have declared that no competing interests exist.

Abstract

Sustained overseas military operations over the last two decades have resulted in large numbers of United States and Allied servicemembers being faced with multiple unique occupational and environmental stressors, from serving in a combat zone, to having to be away from family and home for long periods of time. These stressors result in numerous negative health (physical and mental) and behavioral outcomes. Whereas there is a substantial amount of research focused on deployment-related health outcomes within active duty military populations, reserve forces are less understood. This study focuses on a United States Army National Guard combat unit before, during and after a deployment to Afghanistan. This prospective longitudinal study, conducted over the course of an operational deployment cycle (i.e., before, during and after), documents the trajectories of salient mental health outcomes (i.e., post-traumatic stress, depression, general anxiety, and aggression). The findings show that both combat (e.g., killing others) and non-combat (e.g., boredom) stressors negatively affect mental health outcomes, and the severity of these outcomes increases over the course of a deployment cycle. Of special note, the study reveals key gender differences in the evolution of PTSD, depression and anxiety across a deployment cycle: females report increased PTSD, depression and anxiety 6 months post-deployment whereas the levels reported by males stabilize at their mid-deployment levels. The findings offer insights for medical providers and policymakers in developing more targeted health promotion campaigns and interventions, especially during the post-deployment phase.

Introduction

Trajectories of mental health outcomes across a combat deployment cycle

The United States (US) military and its allies have been engaged in protracted military operations across the globe for nearly two decades, which has resulted in many casualties and

substantial devastation; for instance, during Operations Iraqi Freedom, Enduring Freedom and Freedom's Sentinel, the US military alone has endured ~6,790 total deaths (~4,056 killed in action) and ~52,212 wounded [1]. These operations have in turn resulted in a multitude of mental health (MH) and behavioral health (BH) outcomes [2]. Despite the growth of military population focused research, the underlying factors and full implications of sustained contemporary military operations on the health and well-being of servicemembers are still not comprehensively understood [3]. One limitation in the extant literature is the disproportionate focus on active component (AC; i.e., full-time force) servicemembers, resulting in a knowledge gap concerning the heavily relied upon reserve component (RC; i.e., part-time force).

In the US, the RC comprises the federally controlled Army Reserve (USAR) and the state-controlled National Guard (NG). The NG is a unique and understudied population within the US Army in that it is not only charged with providing most of the RC's combat forces (the USAR's primary focus is support-related) for traditional military operations but it is also charged with supporting state government domestic missions, which range from responding to natural disasters and terrorist attacks to supporting law enforcement during civil unrest [4–5]. As such, within the RC population, the NG is exposed to numerous unique stressors; the result being that these servicemembers often exhibit higher levels of MH issues and compared to their AC/RC peers [6–8] and lack access to treatment options [9–10]. This understudied population warrants research attention to identify risk and protective factors associated with its occupational exposures and enhance its health and well-being needs [11–12].

Key health implications associated with military service

In the US, a large number of servicemembers and veterans continue to suffer MH and maladaptive behavioral issues despite the numerous programs that strive to mitigate the negative outcomes associated with military service [13–14]. Among the MH issues, Posttraumatic Stress Disorder (PTSD) remains a forefront concern and persists in military and veteran populations [15]. Military population PTSD rates are higher than those observed in the general population [16–18]. The core symptoms of PTSD include hyperarousal, avoidance, numbing, and re-experiencing of trauma [19–20]; its etiology is distinctive amongst other MH outcomes in that the defining traumatic event can usually be identified, thus allowing for a more precise cause-and-effect relationship [21–22]. Although PTSD is a key concern within military populations, it is often comorbid with other MH outcomes (e.g., depression and suicidal ideation) as well as problematic substance use [23–24]; for example, those with PTSD are seven times more likely to concurrently suffer from depression compared to those without PTSD [25–26].

Servicemembers face a variety of environmental stressors during combat deployment. One of the most documented stressors is combat experiences (e.g., fighting, killing/injuring others, threat to oneself, exposure to death/injury, and witnessing atrocities) known to trigger negative MH and maladaptive behavioral outcomes (e.g., substance abuse and misuse) [10, 27]. In addition to combat stressors, which can ebb and flow, servicemembers also face a number of more constant deployment-related environmental stressors, which include issues such as boredom, tedious and monotonous workloads, poor food quality, a lack of personal time and space, inability to maintain hygiene, difficulty in maintaining social support relationships with family and friends back home, as well as interpersonal issues with their unit's leadership [28–29, 8]. Although understudied compared to direct combat stressors, such deployment-related environmental stressors are known to increase the prevalence rates of MH and maladaptive behavioral outcomes [30]. A longitudinal approach is needed to provide better insights concerning the relationship between how exposure to deployment-related stressors, combat or otherwise, evolve into MH and maladaptive behavioral issues over the course of a deployment cycle [31].

These issues are particularly salient for RC servicemembers who often struggle with the rapid transition between military and civilian life [32–33]; for instance, reservists returning from a deployment often exhibit increased levels of violent behavior, whether or not they served in a combat role [34] as well as PTSD [35].

Methods

This study utilizes a prospective longitudinal research design with data collected at three time points: pre-, mid- and post-deployment. Prospective longitudinal studies are suited to differentiate between short and long-term influences and serve to establish time-ordered associations between environmental exposures/stressors and health/behavioral outcomes compared to cross-sectional and retrospective studies [36]. This approach enhances the robustness of analyses by allowing an assessment of medical outcome trajectories. Unfortunately, this approach is seldom used in military studies due to logistical difficulties and resource limitations and, to our knowledge, it has not been undertaken before in such a manner with a NG cohort.

Study approval

Study approval was obtained from the Institutional Review Boards (IRB) of the US Department of Defense's (DoD) Uniformed Services University and the US Army Medical Research and Materiel Command. Additionally, as NG personnel fall under the control of the states and territories to which they are assigned, general study approval was also obtained from the National Guard Bureau and the unit's Adjutant General (i.e., the overall commanding officer). For the deployment phase of the study, general study authorization was obtained from North Atlantic Treaty Organization and the US Central Command, which were responsible for servicemembers serving in Afghanistan.

Study population

The study population consists of servicemembers from an infantry brigade (N = 585) from the US Territory of Guam (~48% of the entire Guam Army National Guard) that deployed to Afghanistan between April 2013 and January 2014. Baseline pre-deployment survey data (N = 526; 89.9% of the unit's total) were collected in April 2013 at the unit's mobilization station several days before it deployed to Afghanistan. Data could not be collected from the entire unit prior to deployment as approximately 50 servicemembers had deployed ahead of the main unit to make preparations; however, these servicemembers were given an opportunity to partake in the remaining data collections. Mid-deployment survey data (N = 571; 97.6% of the unit's total) were collected throughout Afghanistan in September and October 2013. Not all servicemembers were available due to mission requirements (e.g., on patrol); additionally, two servicemembers were killed in action prior to the mid-deployment data collection. Post-deployment survey data (N = 472; 80.7% of the unit's total) were collected approximately six months post-deployment, when mental and behavioral health issues often develop [37–38]. Post-deployment data were collected at the unit's primary home base in July and August 2014; however, some data were collected from servicemembers who were receiving medical care at Tripler Army Medical Center, Hawai'i in August 2014.

Informed consent and data collection processes

For all data collections, servicemembers attended a recruitment briefing during routine duty hours that outlined the study's purpose and their rights. During the briefing, servicemembers were encouraged to ask questions. Those wishing to partake in the study were required to

complete an informed consent form at each time point; the consent forms were secured separately from the surveys to help maintain anonymity. Upon submitting their informed consent form, respondents were provided with a paper survey. Respondents were informed that they could skip any questions that made them uncomfortable and cease the study at any time. To help maximize the longitudinal study respondents' confidentiality, surveys were de-identified using an anonymous self-generated sequential code (e.g., PPS1423) as to allow the linkage of individual respondent surveys across the three timepoints. To do so, as in previous military research, respondents listed the last character of their mother's maiden name, day of their birth month, and year of their birth and also the first digits of their birth month and city where they were born [39].

The surveys were matched with probabilistic record linkage based on the self-generated codes [40]. For the purposes of this study, an additional step was taken in that respondents were excluded from the analyses if they did not fully complete all of the MH measure items across all three timepoints. Per IRB guidance, respondents were allowed to skip any survey item that might make them uncomfortable. Ensuring that there was no missing data resulted in a more thorough longitudinal dataset and this conservative approach yielded a total sample of 246 servicemembers with complete measures at each of the three time points.

Survey measurements

Validated measures used in both previous military and civilian studies were administered at each of the three timepoints.

Mental and behavioral health measures. Post-traumatic stress: PTS symptomology was assessed using the 17-item PTSD Checklist (PCL-17; $\alpha = .98$) [17, 41]. The PCL-17 lists all intrusion, avoidance and arousal PTSD symptoms. Respondents rated each item on a 5-point scale (1 = *not at all* to 5 = *extremely)* and the sum of these responses provided an indicator of PTS symptom severity (17-item summation). Respondents with sum scores $\geq$ 50 were considered a positive screen for PTSD [17].

Depression: Depressive symptomology was assessed using the 9-item Patient Health Questionnaire subscale (PHQ-9) [42]. The sum of the 9 items provides an indicator of depression severity with probable major depression defined as endorsing five or more of the nine symptoms present "more than half the days" or "most days" in the past two weeks [43, 42].

Generalized anxiety: Anxiety disorder symptomology was assessed with the 7-item Generalized Anxiety Disorder measure (GAD-7) [44]. The sum provides an indicator of severity and a score $\geq$ 10 was coded as a positive screen for anxiety.

Anger: Anger negatively impacts one's ability to recover from traumatic experiences, especially following a military deployment [45–48]. Recent expressions of internally-focused anger were assessed using a measure adapted from the Interpersonal Conflict and the State/Trait Anger scales for use in military research [49]. Respondents indicated how many times in the past month (1 = *not at all* to 5 = *very often*) they had: boiled inside with anger, a hard time cooling down when angry, anger that got in the way of getting along with others, and anger that progresses instantly to aggression or rage ($\alpha = .93$).

Aggression: Externally-focused aggressive behavior [50] was measured by having respondents indicate how many times in the past month (1 to 10+) they had: been angry at someone and yelled or shouted at them; been angry with someone and kicked or smashed something; slammed a door, punched a wall, etc.; threatened someone with physical violence; and gotten into a fight with someone and hit that person ($\alpha = .83$).

General mental and physical health: Two single-item measures were used to assess respondents' general mental and physical health. Respondents indicated the number of days over the past 30 days when they had poor mental or physical health.

Exposure measures. Deployment stressors: During the mid-deployment data collection, combat- and non-combat deployment stressors were assessed to capture for the degree to which each respondent experienced a number of deployment-related environmental stressors. Combat experiences place servicemembers at particular risk for developing a range of mental and behavioral health issues [17, 14, 10, 27] as well as physical ailments [51, 18]. Combat exposures were evaluated using a 31-item measure that has been used in multiple military studies (α = .78) [17, 10, 52]; respondents were asked to indicate how many times during their current deployment they had experienced each item (e.g., Being shot at; 0 = 0 to 6 = 5+). Non-combat deployment stressors were assessed with a 25-item measure created for the purposes of this study; respondents were asked to indicate how stressful (0 = *not at all* to 5 = *extremely*) each item had been on their current deployment (α = .95). See supplemental information for a complete list of the items.

Additional variables. A number of factors known to impact both the onset and treatment of MH outcomes were also assessed in the post-deployment survey.

Stigma and barriers to care: Perceived stigmas associated with seeking MH care were assessed in the post-deployment survey with a 10-item measure (e.g., I do not trust mental health professionals; α = .96) [53–54]. Barriers to utilizing care were assessed with a 6-item measure (e.g., not knowing where to get help from; α = .92). Respondents were asked to "rate each of the possible concerns that might have affected your decision whether or not to receive mental health counseling or services during the past month" (1 = *strongly disagree* to 5 = *strongly agree*) [53–54]. See supplemental information for a complete list of the items.

Organizational support: The degree to which respondents received positive psychological support from their unit overall was assessed with the shortened Perceived Organizational Support measure, which has been widely used in military research [55–56]; respondents indicated their level of agreement with the following items: 1) My unit strongly considers my goals and values; 2) My unit really cares about my well-being; 3) My unit cares about my opinion; and 4) My unit is willing to help me when I need a special favor (0 = *strongly disagree* to 5 = *strongly agree*; α = .95).

Unit cohesion: The degree to which respondents perceived their immediate unit as functioning cohesively was assessed by asking respondents to indicate their level of agreement with the following statements: 1) The members of my unit are cooperative with each other; 2) The members of my unit know that they can depend on each other; and 3) The members of my unit stand up for each other (0 = *strongly disagree* to 5 = *strongly agree*; α = .96) [57–58].

Facets of perceived leadership: Respondents' perceptions of their immediate leadership in their unit (e.g., those directly in charge of them) were assessed by respondents indicating how often leaders did the following: 1) Tell servicemembers when they have done a good job; 2) Embarrass servicemembers in front of other servicemembers; 3) Try to look good to higher-ups by assigning extra missions or details to servicemembers; 4) Exhibit clear thinking and reasonable action under stress. Each of these items was measured from 0 = *never* to 5 = *always*) [59–61].

Reintegration: General post-deployment reintegration was assessed with a shortened 11-item version of the Military to Civilian Questionnaire [62]; respondents indicated the level of difficulty they had with each item (e.g., Finding meaning or purpose in life) since returning from the deployment (0 = *no difficulty* to 5 = *extreme difficulty*; α = .95). Additionally, post-deployment family reintegration was assessed with a shortened 10-item Army Post-Deployment Reintegration Scale [63]; respondents indicated the extent to which each item (e.g., I feel closer to my family) held true for them since returning from deployment (0 = *not true at all* to 5 = *completely true*; α = .78). See supplemental information for a complete list of the items.

Demographics: Consistent with other military studies, the key variables included: gender, military rank, age, ethnicity, education level, marital or significant other status, and years of military service. As this population is comprised of reserve personnel, more civilian-centric

questions were also posed, including: civilian employment status (full-time, part-time, unemployed, retired), employment type (self-employed, government employee, or private sector employee) socioeconomic status (annual household income and debt level), and college/university student status (full or part-time). No personally identifiable information was collected (e.g., birthdate).

Results

Sample demographics

The analysis sample consisted of 246 respondents with matched and complete MH data across the three time points. The majority were male (90.7%), Pacific Islander (84.6%), Chamorro (i.e., native people of Guam; 80.80%). Ages ranged between 19 and 57 years at mid- deployment, with a mean age of 29.50. The demographic characteristics, rank, education levels, marital status and deployment history of this matched sample are similar to those of the complete population (the whole unit who deployed), as reported in Table 1.

Mental health outcomes

Table 2 provides the scores for each MH outcome of interest at the three timepoints and, where applicable, the total number and percentage of servicemembers who screened positive at each timepoint.

Table 1. Demographic characteristics of the analysis sample (matched data across time points) with population.

	Population (Unit)	Analysis Sample
**Demographics (as reported mid-deployment)**		
N	571	246
Gender N males (% of total reported)	521 (91.7%)	223 (90.7%)
Ethnicity N Pacific Islander (% of total reported)	450 (82.0%)	208 (84.6.%)
Age Mean (SD)	29.18 (8.52)	29.50 (8.23)
**Rank**		
E1-E3	37 (4.7%)	18 (7.3%)
E4	298 (37.5%)	143 (58.1%)
E5-E6	154 (19.4%)	62 (25.2%)
E7-E9	34 (6.0%)	9 (3.7%)
Officer / Warrant Officer	46 (5.8%)	14 (5.7%)
**Education** (as reported pre deployment)		
< 12th grade	10 (1.9%)	6 (2.5%)
High School diploma / GED	267 (51.9%)	128 (52.7%)
Some college / technical school	190 (37%)	91 (37.4%)
Bachelor’s degree	37 (7.2%)	15 (6.2%)
Graduate degree	10 (1.9%)	3 (1.2%)
**Marital Status** (as reported pre deployment)		
Single never married	195 (37.4%)	97 (39.6%)
Married	294 (56.3%)	135 (55.1%)
Divorced or separated	33 (6.3%)	13 (5.3%)
**Deployment History** (as reported pre deployment) N	316	154
0 previous deployment	257 (81.3%)	131 (85.1%)
1 previous deployment	32 (10.1%)	11 (7.1%)
2 + previous deployments	27 (8.6%)	12 (7.8%)

https://doi.org/10.1371/journal.pone.0223855.t001

Table 2. Mental health outcomes across time points (N = 246).

	Range of scores/scale	Pre-deployment	Mid-deployment	Post-deployment
**Mental Health Outcomes**				
**Post-Traumatic Stress Disorder**				
PCL-17 Sum Score (SD)	17–79	22.82 (9.73)	27.34 (12.29)	27.75 (14.36)
PTSD Positive screen N (%)		12 (4.9%)	28 (11.4%)	30 (12.2%)
**Depression**				
PHQ-9 sum score (SD)	0–27	1.41 (2.94)	2.69 (4.37)	2.98 (4.92)
Depression Positive screen		5 (2.0%)	7 (2.8%)	25 (10.2%)
**General Anxiety Disorder**				
GAD-7 Sum Score (SD)	0–14	2.15 (2.77)	2.70 (3.17)	3.00 (3.65)
Anxiety positive screen N (%)		6 (2.5%)	7 (2.8%)	17 (7.1%)
**Internal Anger**	1–5	1.37 (.61)	1.60 (.85)	1.67 (.96)
**Aggression**	0–10	.55 (1.33)	.60 (1.27)	1.34 (1.69)
**Bad Mental Health days (# in last 30 days)**	0–30	.22 (1.71)	.84 (3.72)	1.34 (5.04)
**Bad Physical Health days (# in last 30 days)**	0–30	.63 (2.45)	1.34 (4.01)	2.38 (6.90)

https://doi.org/10.1371/journal.pone.0223855.t002

To assess the trajectories over the three timepoints and the role of combat and deployment-related stress factors, as well as address demographic differences in those trajectories, a series of repeated measures regression analyses were conducted: each of the continuous MH outcomes was analyzed with time as a repeated measure (3 timepoints) and included in the model main effects for gender, age, combat experiences and deployment stressors as well as their interactions with time. The analysis provides an assessment of the trajectory of the MH across time (main effect of time) as well as whether these trajectories differ significantly as a function of the other variables (interaction between time and the other variables in the model).

As reported in Table 3, time was significant for PCL-17, GAD-7, and PHQ-9, indicating that these MH outcomes worsen over time, which is in line with the scores reported in Table 2. In terms of main effects, combat experiences and deployment-related stressors are both significant predictors of all the MH outcomes, and age is related to PCL-17 and PHQ-9.

Although there are no main effects of gender, the interaction between time and gender is significant for PCL-17, GAD-7, and PHQ-9, signaling that the temporal trajectories of these MH issues differ by gender. These trajectories are illustrated in Figs 1, 2 and 3; whereas scores on the PCL-17, GAD-7, and PHQ-9 assessments stabilize around mi-deployment levels for

Table 3. Repeated measures regression results for each mental health outcome.

F values	PCL-17	PHQ-9	GAD-7	Internal Anger	Aggression
Time	3.70*	6.02**	5.66**	.77	2.03
Gender	.47	1.92	.56	.31	.87
Age	9.03**	5.55*	2.67	.84	.95
Combat Experiences	19.70**	8.44**	21.65**	11.59**	14.82**
Deployment Stressors	55.60**	70.25**	67.08**	62.62**	23.85**
Time X Gender	3.62*	3.26*	7.11**	2.72	.97
Time X Age	1.29	4.27*	2.62	.52	1.08
Time X Combat Experiences	.29	.71	.01	1.05	4.32*
Time X Deployment Stressors	15.60**	14.81**	9.14**	6.31**	.95

Note: *$p < .05$;

**$p < .01$.

https://doi.org/10.1371/journal.pone.0223855.t003

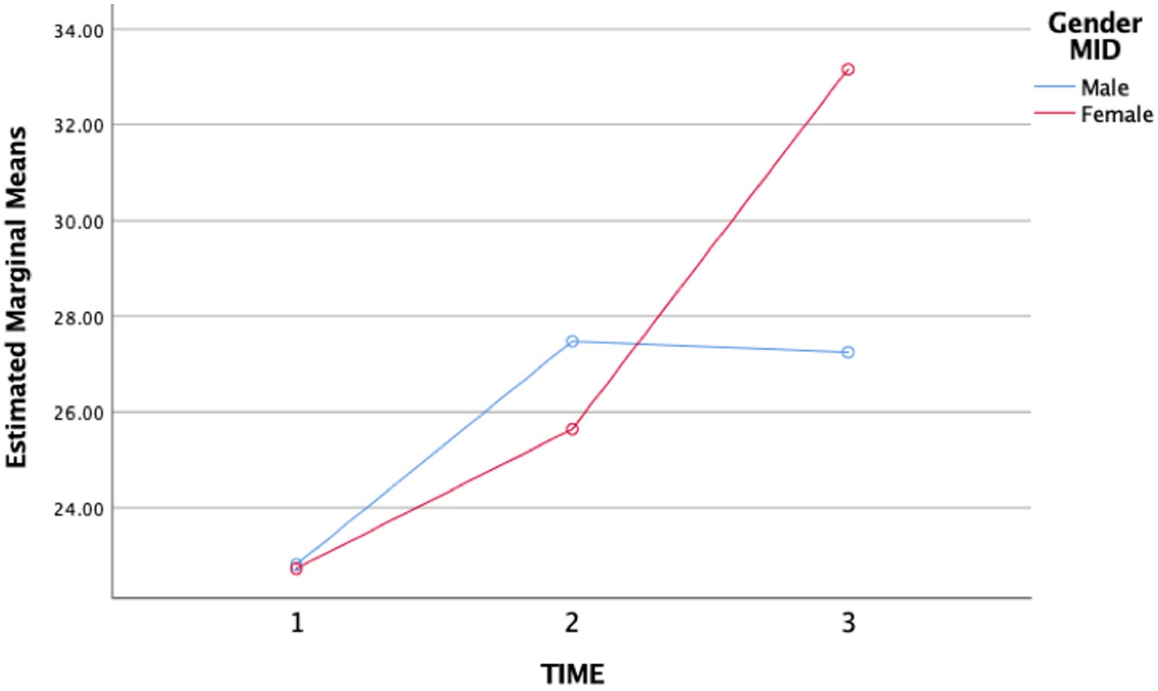

Fig 1. Estimated marginal means, PCL-17 across time: Males vs. Females.

https://doi.org/10.1371/journal.pone.0223855.g001

males, they are all significantly higher for females than for males six months post-deployment. Table 4 shows that female respondents report higher levels of post-deployment MH outcomes,

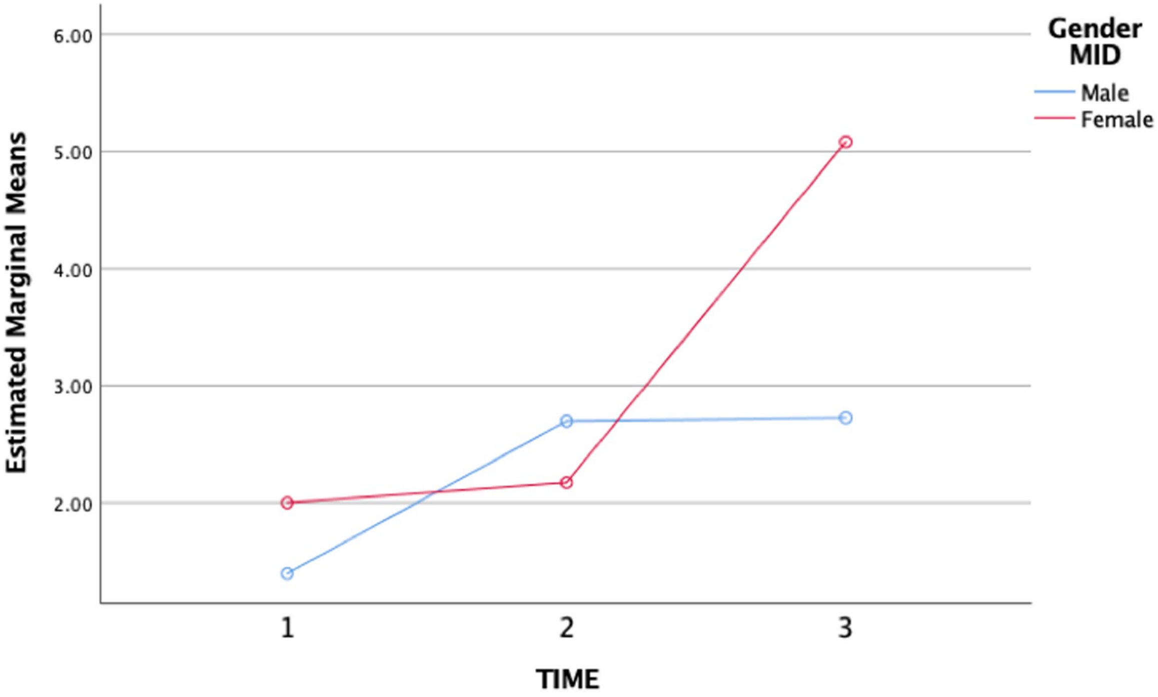

Fig 2. Estimated marginal means, PHQ-9 across time: Males vs. Females.

https://doi.org/10.1371/journal.pone.0223855.g002

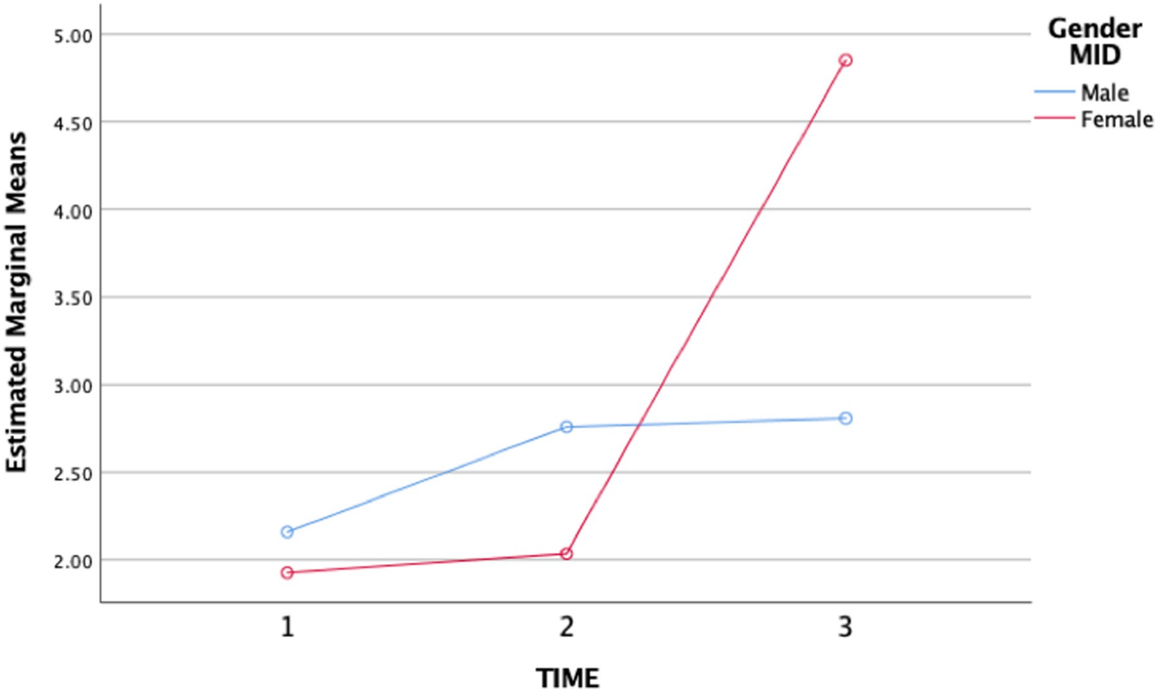

Fig 3. Estimated marginal means, GAD-7 across time: Males vs. Females.

https://doi.org/10.1371/journal.pone.0223855.g003

even though there were no such gender differences before or during deployment. These analyses control for combat and non-combat deployment stressors, so the gender differences identified are independent of those factors, which are also significantly and positively associated with MH outcomes.

Exploratory post analysis

Given the significant gender differences in the MH trajectories across the combat deployment cycle, an additional exploratory analysis was conducted to better understand the nature of the situation females face post-deployment. Focusing in on the post-deployment data and comparing gender in an independent sample t-test between the two groups, females were found not to differ from their male counterparts in terms of post-deployment reintegration, family reintegration,

Table 4. Gender differences in mental health outcomes across time points (if time X gender is significant).

	Pre-deployment	Mid-deployment	Post-deployment
**Post-Traumatic Stress Disorder**			
PCL-17 Sum Score (SD)—Males	22.80 (9.73)	27.67 (12.58)	27.39 (14.46)
PCL-17 Sum Score (SD)—Females	21.47 (6.39)	23.71 (7.95)	31.65 (13.39)**
**Depression**			
PHQ-9 Sum Score (SD)—Males	1.43 (2.95)	2.74 (4.36)	2.76 (4.82)
PHQ-9 Sum Score (SD)—Females	1.62 (3.56)	1.76 (3.19)	4.76 (5.43)**
**General Anxiety Disorder**			
GAD-7 Sum Score (SD)—Males	2.20 (2.83)	2.81 (3.25)	2.85 (3.59)
GAD-7 Sum Score (SD)—Females	1.52 (1.99)	1.52 (1.91)	4.43 (4.08)**

Note: **$p < .01$.

https://doi.org/10.1371/journal.pone.0223855.t004

barriers to care or stigma of care. The only factors that differed significantly between males and females were those related to the organization support received (or not) from the unit, with females reporting significantly lower rates of organization support (3.27 for females vs. 3.88 for males), or unit cohesion (3.54 for females vs. 4.02 for males) post- deployment (even though there were no such gender differences at mid-deployment; all post- deployment *t*s $(244) > 2.65$, p $< .05$). Two items in the perceived support from leadership differ by gender: females were significantly less likely to report that unit leadership told servicemembers when they have done a good job (females 3.26 vs. males 3.95; $t\,(242) = 3.63$, $p < .05$), and that unit leadership exhibits clear thinking and reasonable action under stress (females 3.22 vs. males 3.77; $t\,(242) = 3.37$, $p < .05$).

Discussion

This study reveals that the trajectory of MH issues varies as a function of the type of stressor (i.e., both deployment and combat related) experienced across a deployment cycle as well as one's individual characteristics. Most notably, the findings highlight salient gender differences in the trajectories of PTSD, depression and anxiety. Whereas females reported lower rates of MH issues before and during their deployment, their MH outcomes post-deployment are more severe than male counterparts'.

This finding adds to a growing body of research concerning gender-associated health outcome differences in military populations and highlights the need for additional gender-focused research [64]. To that end, an interesting avenue for future research might lie in more deeply documenting the factors, such as social support from fellow unit members, that may be lacking in the post-deployment environment for female servicemembers. Considering that the females in this study reported higher rates of PTSD, depression and anxiety as well as perceptions of receiving less unit and leadership support post-deployment, it is important that future research delineate what factors might underlie such perceptions and how to best palliate these cultural and organizational issues. Within such a line of research, a broader scope of inquiry is required to capture everyday realities post-deployment. It is well-documented that females face hardships and MH issues when returning to work following maternity leave. Research in this realm has shown the importance of accounting for socioeconomic status, childcare responsibilities, and household obligations [65]; and there have been calls for military researchers to better assess specific demographic, social, and environmental exposure factors [66]. Future research should increase focus to such factors when studying military reserve populations and assess the parallels between females returning from military deployments and returning to employment from parental leave. More specifically, future research should seek to understand and develop female-centric post-deployment reintegration and health promotion programs in an effort to mitigate negative MH outcomes.

Although most of the extant literature has focused on combat-related environmental stressors, this study's findings suggest that more attention should be placed on everyday deployment-related stressors, which emerged as significant drivers of MH issues. Compared to combat-related stressors, non-combat stressors span a large spectrum and range from less serious factors, such as boredom, to more serious factors, such as sexual harassment [67, 30]. As to better account for the multitude of environmental stressors that servicemembers face on a deployment, future research should more accurately identify, operationalize and account for non-combat deployment-related stressors as to assess the degree to which they negatively impact health and well-being outcomes across a deployment cycle for active duty and reserve servicemembers alike.

In closing, we acknowledge that the study's participants were primarily "Native Hawaiian or Other Pacific Islander." While the focus on servicemembers from Guam may not generalize to the wider US or other military forces, this population represents a unique and understudied

segment of the military. Given that this isolated island nation is known to harbor strong cultural identities and social bonds, future research on aspects of the sociocultural environment that might afford protective factors could inform future research and culturally relevant prevention and intervention efforts in the area of mental health.

Supporting information

S1 File. Deidentified minimal dataset used in the MLM analyses.
(XLSX)

Acknowledgments

The authors express their gratitude to the North Atlantic Treaty Organization Training Mission in Afghanistan and the US Central Command for supporting this research project. The authors would like to dedicate this paper to SGT Eugene M. Aguon and SPC Dwayne W. Flores, who were part of the unit understudy and killed in action in Afghanistan on 16 May 2013 during the course of the research. The views expressed are those of the authors and do not reflect the official policy or position of the US Government, the US Department of Defense, or the Uniformed Services University.

Author Contributions

Data curation: Dale W. Russell, Cristel Antonia Russell.

Formal analysis: Dale W. Russell, Cristel Antonia Russell.

Investigation: Dale W. Russell, Cristel Antonia Russell.

Methodology: Dale W. Russell, Cristel Antonia Russell.

Project administration: Dale W. Russell.

Resources: Dale W. Russell.

Supervision: Dale W. Russell.

Validation: Dale W. Russell, Cristel Antonia Russell.

Writing – original draft: Dale W. Russell, Cristel Antonia Russell.

Writing – review & editing: Dale W. Russell, Cristel Antonia Russell.

References

1. DeBruyne NF, Leland A. American war and military operations casualties: Lists and statistics. Congressional Research Service Washington United States; 2015 Jan 2. Available from: https://fas.org/sgp/crs/natsec/RL32492.pdf.
2. Ciarleglio MM, Aslan M, Proctor SP, Concato J, Ko J, Kaiser AP, et al. Associations of stress exposures and social support with long-term mental health outcomes among US Iraq War Veterans. Behav Ther. 2018 Sep 1; 49(5):653–67. https://doi.org/10.1016/j.beth.2018.01.002 PMID: 30146134
3. Yang S, Wynn GH, Ursano RJ. A clinician's guide to PTSD biomarkers and their potential future use. Focus. 2018 Apr 27; 16(2):143–52.
4. Fink DS, Calabrese JR, Liberzon I, Tamburrino MB, Chan P, Cohen GH, et al. Retrospective age-of-onset and projected lifetime prevalence of psychiatric disorders among US Army National Guard soldiers. J Affect Disorders. 2016 Sep 15; 202:171–7. https://doi.org/10.1016/j.jad.2016.05.025 PMID: 27262639
5. Russell DW, Kazman JB, Benedek DM, Ursano RJ, Russell CA. Domestic Civil Support Missions Can Aggravate Negative Mental Health Outcomes Among National Guardsmen: The Moderating Role of Economic Difficulties. J Trauma Stress. 2017 Apr; 30(2):195–9. https://doi.org/10.1002/jts.22164 PMID: 28141895

6. Hofscher R, Bennett E, Crabtree M, Russell E, Isacco A. National Guard and Reserve: An Examination of Differences on Posttraumatic Stress Symptoms. Mil Behav Health. 2017 Apr 3; 5(2):157–62.
7. Russell DW, Cohen GH, Gifford R, Fullerton CS, Ursano RJ, Galea S. Mental health among a nationally representative sample of United States Military Reserve Component Personnel. Soc Psychiatry Psychiatr Epidemiol. 2015 Apr 1; 50(4):639–51. https://doi.org/10.1007/s00127-014-0981-2 PMID: 25421591
8. Russell DW, Benedek DM, Naifeh JA, Fullerton CS, Benevides N, Ursano RJ, et al. Social support and mental health outcomes among US Army Special Operations personnel. Mil Psychol. 2016 Oct 6; 28 (6):361–75.
9. Bennett EA, Crabtree M, Schaffer ME, Britt TW. Mental health status and perceived barriers to seeking treatment in rural reserve component veterans. JRSS. 2011 Sep 1; 26(3):113.
10. Russell DW, Russell CA, Riviere LA, Thomas JL, Wilk JE, Bliese PD. Changes in alcohol use after traumatic experiences: The impact of combat on Army National Guardsmen. Drug Alcohol Depend. 2014 Jun 1; 139:47–52. https://doi.org/10.1016/j.drugalcdep.2014.03.004 PMID: 24685562
11. Heavey SC, Homish DL, Goodell EA, Homish GG. US reserve soldiers' combat exposure and intimate partner violence: Not more common but it is more violent. Stress Health. 2017 Dec; 33(5):617–23. https://doi.org/10.1002/smi.2748 PMID: 28198140
12. Hoopsick RA, Vest BM, Homish DL, Homish GG. Combat exposure, emotional and physical role limitations, and substance use among male United States Army Reserve and National Guard soldiers. Qual Life Res. 2018 Jan 1; 27(1):137–47. https://doi.org/10.1007/s11136-017-1706-2 PMID: 28921407
13. Defense Health Agency. Mental health disorder prevalence among active duty service members in the military health system, fiscal years 2005–2016. Silver Spring, MD: Deployment Health Clinical Center, Defense Centers of Excellence for Psychological Health and Traumatic Brain Injury Center. 2017.
14. Norman SB, Haller M, Hamblen JL, Southwick SM, Pietrzak RH. The burden of co-occurring alcohol use disorder and PTSD in US Military veterans: Comorbidities, functioning, and suicidality. Psychol Addict Behav. 2018 Mar; 32(2):224. https://doi.org/10.1037/adb0000348 PMID: 29553778
15. Ramchand R, Rudavsky R, Grant S, Tanielian T, Jaycox L. Prevalence of, risk factors for, and consequences of posttraumatic stress disorder and other mental health problems in military populations deployed to Iraq and Afghanistan. Current psychiatry reports. 2015 May 1; 17(5):37. https://doi.org/10.1007/s11920-015-0575-z PMID: 25876141
16. Broekman BF, Olff M, Boer F. The genetic background to PTSD. Neurosci Biobehav Rev. 2007 Jan 1; 31(3):348–62. https://doi.org/10.1016/j.neubiorev.2006.10.001 PMID: 17126903
17. Hoge CW, Castro CA, Messer SC, McGurk D, Cotting DI, Koffman RL. Combat duty in Iraq and Afghanistan, mental health problems, and barriers to care. N Eng J Med. 2004 Jul 1; 351(1):13–22.
18. Hoge CW, Terhakopian A, Castro CA, Messer SC, Engel CC. Association of posttraumatic stress disorder with somatic symptoms, health care visits, and absenteeism among Iraq war veterans. Am J Psychiatry. 2007 Jan; 164(1):150–3. https://doi.org/10.1176/ajp.2007.164.1.150 PMID: 17202557
19. American Psychiatric Association. Diagnostic and statistical manual of mental disorders (DSM-5). American Psychiatric Pub; 2013 May 22.
20. Yehuda R. Advances in understanding neuroendocrine alterations in PTSD and their therapeutic implications. Ann N Y Acad Sci. 2006 Jul 1; 1071(1):137–66.
21. Koenen KC, Saxe G, Purcell S, Smoller JW, Bartholomew D, Miller A, et al. Polymorphisms in FKBP5 are associated with peritraumatic dissociation in medically injured children. Mol Psychiatry. 2005 Aug 9; 10(12):1058. https://doi.org/10.1038/sj.mp.4001727 PMID: 16088328
22. Koenen KC, Stellman SD, Sommer JF Jr, Stellman JM. Persisting posttraumatic stress disorder symptoms and their relationship to functioning in Vietnam veterans: a 14-year follow-up. J Trauma Stress. 2008 Feb; 21(1):49–57. https://doi.org/10.1002/jts.20304 PMID: 18302174
23. Odani S, Agaku IT, Graffunder CM, Tynan MA, Armour BS. Tobacco product use among military veterans—United States, 2010–2015. MMWR. 2018 Jan 12; 67(1):7. https://doi.org/10.15585/mmwr.mm6701a2 PMID: 29324732
24. Olenick M, Flowers M, Diaz VJ. US veterans and their unique issues: enhancing health care professional awareness. Adv Med Educ Pract. 2015; 6:635. https://doi.org/10.2147/AMEP.S89479 PMID: 26664252
25. Breslau N, Davis GC, Peterson EL, Schultz LR. A second look at comorbidity in victims of trauma: The posttraumatic stress disorder–major depression connection. Biol Psychiatry. 2000 Nov 1; 48(9):902–9. https://doi.org/10.1016/s0006-3223(00)00933-1 PMID: 11074228
26. McFarlane AC, Papay P. Multiple diagnoses in posttraumatic stress disorder in the victims of a natural disaster. J Nerv Ment Dis. 1992 Aug; 180(8):498–504. https://doi.org/10.1097/00005053-199208000-00004 PMID: 1500931

27. Russell DW, Whalen RJ, Riviere LA, Clarke-Walper K, Bliese PD, Keller DD, et al. Embedded behavioral health providers: An assessment with the Army National Guard. Psychol Serv. 2014 Aug; 11 (3):265. https://doi.org/10.1037/a0037005 PMID: 24841511
28. Booth-Kewley S, Larson GE, Highfill-McRoy RM, Garland CF, Gaskin TA. Correlates of posttraumatic stress disorder symptoms in Marines back from war. J Trauma Stress. 2010 Feb; 23(1):69–77. https://doi.org/10.1002/jts.20485 PMID: 20104587
29. Halverson RR, Bliese PD, Moore RE, Castro CA. Psychological Well-Being and Physical Health Symptoms of Soldiers Deployed for Operation Uphold Democracy. A Summary of Human Dimensions Research in Haiti (No. WRAIR/TR-95-0008). Washington DC: Walter Reed Army Inst of Research. 1995 May 25.
30. Mayeux R, Basham KK, Bromet EJ, Burke GL, Charney DS, Davis M, et al. Gulf War and Health. 2008;6. Available from: https://www.nap.edu/read/11922.
31. Linley PA, Joseph S. Positive change following trauma and adversity: A review. J Trauma Stress. 2004 Feb; 17(1):11–21. https://doi.org/10.1023/B:JOTS.0000014671.27856.7e PMID: 15027788
32. Morden E, Oster M, O'Brien CP, editors. Substance use disorders in the US Armed Forces. National Academies Press; 2013 Mar 21.
33. Schaller EK, Woodall KA, Lemus H, Proctor SP, Russell DW, Crum-Cianflone NF. A longitudinal comparison of posttraumatic stress disorder and depression among military service components. Mil Psychol. 2014 Mar 1; 26(2):77–87.
34. Kwan J, Jones M, Hull L, Wessely S, Fear N, MacManus D. Violent behavior among military reservists. Aggress Behav. 2017 May; 43(3):273–80. https://doi.org/10.1002/ab.21687 PMID: 27775160
35. Polusny MA, Martyr MA, Erbes CR, Arbisi PA, Kramer M, Gibson E, et al. Prevalence and Risk Factors for Post-Traumatic Stress Disorder Symptoms among National Guard/Reserve Component Service Members Deployed to Iraq and Afghanistan. Comprehensive Guide to Post-Traumatic Stress Disorders. 2016 Jun:455–87.
36. Pietrzak E, Pullman S, Cotea C, Nasveld P. Effects of deployment on mental health in modern military forces: A review of longitudinal studies. J Mil Veterans Health. 2012 Aug; 20(3):24.
37. Bliese PD, Wright KM, Adler AB, Thomas JL, Hoge CW. Timing of postcombat mental health assessments. Psychol Serv. 2007 Aug; 4(3):141.
38. Thomas JL, Wilk JE, Riviere LA, McGurk D, Castro CA, Hoge CW. Prevalence of mental health problems and functional impairment among active component and National Guard soldiers 3 and 12 months following combat in Iraq. Arch Gen Psychiatry. 2010 Jun 1; 67(6):614–23. https://doi.org/10.1001/archgenpsychiatry.2010.54 PMID: 20530011
39. Wilson AL, Hoge CW, McGurk D, Thomas JL, Clark JC, Castro CA. Application of a new method for linking anonymous survey data in a population of soldiers returning from Iraq. Ann Epidemiol. 2010 Dec 1; 20(12):931–8. https://doi.org/10.1016/j.annepidem.2010.08.008 PMID: 21074108
40. Grube JW, Morgan M, Kearney KA. Using self-generated identification codes to match questionnaires in panel studies of adolescent substance use. Addict Behav. 1989 Jan 1; 14(2):159–71. https://doi.org/10.1016/0306-4603(89)90044-0 PMID: 2786325
41. Weathers FW, Litz BT, Herman DS, Huska JA, Keane TM. The PTSD Checklist (PCL): Reliability, validity, and diagnostic utility. San Antonio, TX: Annual convention of the international society for traumatic stress studies. 1993 Oct 24;462.
42. Spitzer RL, Kroenke K, Williams JB, Patient Health Questionnaire Primary Care Study Group. Validation and utility of a self-report version of PRIME-MD: the PHQ primary care study. JAMA. 1999 Nov 10; 282(18):1737–44. https://doi.org/10.1001/jama.282.18.1737 PMID: 10568646
43. Lang AJ, Laffaye C, Satz LE, Dresselhaus TR, Stein MB. Sensitivity and specificity of the PTSD checklist in detecting PTSD in female veterans in primary care. J Trauma Stress. 2003 Jun; 16(3):257–64. https://doi.org/10.1023/A:1023796007788 PMID: 12816338
44. Spitzer RL, Kroenke K, Williams JB, Löwe B. A brief measure for assessing generalized anxiety disorder: the GAD-7. Arch Intern Med. 2006 May 22; 166(10):1092–7. https://doi.org/10.1001/archinte.166.10.1092 PMID: 16717171
45. Adler AB, Britt TW, Castro CA, McGurk D, Bliese PD. Effect of transition home from combat on risk-taking and health-related behaviors. J Trauma Stress. 2011 Aug; 24(4):381–9. https://doi.org/10.1002/jts.20665 PMID: 21818784
46. Ehlers A, Mayou RA, Bryant B. Psychological predictors of chronic posttraumatic stress disorder after motor vehicle accidents. J Abnorm Psychol. 1998 Aug; 107(3):508. https://doi.org/10.1037//0021-843x.107.3.508 PMID: 9715585

47. Forbes D, Bennett N, Biddle D, Crompton D, McHugh T, Elliott P, et al. Clinical presentations and treatment outcomes of peacekeeper veterans with PTSD: Preliminary findings. Am J Psychiatry. 2005 Nov 1; 162(11):2188–90. https://doi.org/10.1176/appi.ajp.162.11.2188 PMID: 16263866

48. McHugh T, Forbes D, Bates G, Hopwood M, Creamer M. Anger in PTSD: is there a need for a concept of PTSD-related posttraumatic anger?. Clin Psychol Rev. 2012 Mar 1; 32(2):93–104. https://doi.org/10.1016/j.cpr.2011.07.013 PMID: 22236575

49. Wilk JE, Bliese PD, Thomas JL, Wood MD, McGurk D, Castro CA, et al. Unethical battlefield conduct reported by soldiers serving in the Iraq war. J Nerv Ment Dis. 2013 Apr 1; 201(4):259–65. https://doi.org/10.1097/NMD.0b013e318288d302 PMID: 23538969

50. Kulka RA, Schlenger WE, Fairbank JA, Hough RL, Jordan BK, Marmar CR, et al. Trauma and the Vietnam war generation: Report of findings from the National Vietnam Veterans Readjustment Study. New York: Brunner/Mazel; 1990 Mar.

51. Boscarino JA. Diseases among men 20 years after exposure to severe stress: implications for clinical research and medical care. Psychosom Med. 1997 Nov 1; 59(6):605–14. https://doi.org/10.1097/00006842-199711000-00008 PMID: 9407579

52. Wilk JE, Bliese PD, Kim PY, Thomas JL, McGurk D, Hoge CW. Relationship of combat experiences to alcohol misuse among US soldiers returning from the Iraq war. Drug Alcohol Depend. 2010 Apr 1; 108 (1–2):115–21. https://doi.org/10.1016/j.drugalcdep.2009.12.003 PMID: 20060237

53. Kim PY, Britt TW, Klocko RP, Riviere LA, Adler AB. Stigma, negative attitudes about treatment, and utilization of mental health care among soldiers. Mil Psychol. 2011 Jan 13. https://doi.org/10.1080/08995605.2011.534407 PMID: 25324594

54. Kim PY, Thomas JL, Wilk JE, Castro CA, Hoge CW. Stigma, barriers to care, and use of mental health services among active duty and National Guard soldiers after combat. Psychiatr Serv. 2010 Jun; 61 (6):582–8. https://doi.org/10.1176/ps.2010.61.6.582 PMID: 20513681

55. Anderson L, Campbell-Sills L, Ursano RJ, Kessler RC, Sun X, Heeringa SG, et al. Prospective associations of perceived unit cohesion with postdeployment mental health outcomes. Depress Anxiety. 2019 Jan 29. Available from: https://onlinelibrary.wiley.com/doi/abs/10.1002/da.22884

56. Kelley CL, Britt TW, Adler AB, Bliese PD. Perceived organizational support, posttraumatic stress disorder symptoms, and stigma in soldiers returning from combat. Psychol Serv. 2014 May; 11(2):229. https://doi.org/10.1037/a0034892 PMID: 24364593

57. Ehrhart MG, Bliese PD, Thomas JL. Unit-level OCB and unit effectiveness: Examining the incremental effect of helping behavior. Hum Perform. 2006 Apr 1; 19(2):159–73.

58. Wright KM, Cabrera OA, Bliese PD, Adler AB, Hoge CW, Castro CA. Stigma and barriers to care in soldiers postcombat. Psychol Serv. 2009 May; 6(2):108.

59. Chen G, Bliese PD. The role of different levels of leadership in predicting self-and collective efficacy: Evidence for discontinuity. J Appl Psychol. 2002 Jun; 87(3):549. https://doi.org/10.1037/0021-9010.87.3.549 PMID: 12090612

60. Thomas JL, Bliese PD, Jex SM. Interpersonal Conflict and Organizational Commitment: Examining Two Levels of Supervisory Support as Multilevel Moderators 1. J Appl Soc Psychol. 2005 Nov; 35(11):2375–98.

61. Zinzow HM, Britt TW, Pury CL, Raymond MA, McFadden AC, Burnette CM. Barriers and facilitators of mental health treatment seeking among active-duty army personnel. Mil Psychol. 2013 Sep 30; 25 (5):514–35.

62. Sayer NA, Frazier P, Orazem RJ, Murdoch M, Gravely A, Carlson KF, et al. Military to civilian questionnaire: A measure of postdeployment community reintegration difficulty among veterans using Department of Veterans Affairs medical care. J Trauma Stress. 2011 Dec; 24(6):660–70. https://doi.org/10.1002/jts.20706 PMID: 22162082

63. Blais AR, Thompson MM, McCreary DR. The development and validation of the army post-deployment reintegration scale. Mil Psychol. 2009 Jun 30; 21(3):365–86.

64. Cohen GH, Sampson LA, Fink DS, Wang J, Russell D, Gifford R, et al. Gender, position of authority, and the risk of depression and posttraumatic stress disorder among a national sample of US Reserve component personnel. Women's Health Issues. 2016 May 1; 26(3):268–77. https://doi.org/10.1016/j.whi.2016.01.001 PMID: 26899583

65. Wallace M, Saurel-Cubizolles MJ, EDEN Mother–Child Cohort Study Group. Returning to work one year after childbirth: data from the mother–child cohort EDEN. Matern Child Health J. 2013 Oct 1; 17 (8):1432–40. https://doi.org/10.1007/s10995-012-1147-z PMID: 23054452

66. Ford JD. Complex PTSD: Research directions for nosology/assessment, treatment, and public health. Eur J Psychotraumatol. 2015 Dec 1; 6(1):27584.

67. Hosek J, Kavanagh JE, Miller LL. How deployments affect service members. Santa Monica, CA: Rand Corporation; 2006 Feb 7.
